# Chemical Stability of Graphene Coated Silver Substrates for Surface-Enhanced Raman Scattering

**DOI:** 10.1038/s41598-017-14782-2

**Published:** 2017-11-01

**Authors:** Seiya Suzuki, Masamichi Yoshimura

**Affiliations:** Graduate School of Engineering, Toyota Technological Institute, 2-12-1 Hisakata, Tempaku, Nagoya, 468-8511 Japan

## Abstract

Surface enhanced Raman spectroscopy (SERS) is a novel method to sense molecular and lattice vibrations at a high sensitivity. Although nanostructured silver surface provides intense SERS signals, the silver surface is unstable under acidic environment and heated environment. Graphene, a single atomic carbon layer, has a prominent stability for chemical agents, and its honeycomb lattice completely prevents the penetration of small molecules. Here, we fabricated a SERS substrate by combining nanostructured silver surface and single-crystal monolayer graphene (G-SERS), and focused on its chemical stability. The G-SERS substrate showed SERS even in concentrated hydrochloric acid (35–37%) and heated air up to 400 °C, which is hardly obtainable by normal silver SERS substrates. The chemically stable G-SERS substrate posesses a practical and feasible application, and its high chemical stability provides a new type of SERS technique such as molecular detections at high temperatures or in extreme acidic conditions.

## Introduction

Surface-enhanced Raman spectroscopy (SERS) is a novel method to sense molecular and lattice vibrations^1–3^. The origin of the intense Raman signals in SERS is enhancement of local electromagnetic fields via the localized surface plasmon resonance effect^4^ in nanostructured metal surface or metal colloids. The main contribution of SERS enhancement is electromagnetic (EM) enhancement up to 10^10^ times^5–7^, and another minor contribution is chemical enhancement including the resonance Raman effect, the charge-transfer effect, and the adsorption effect^8^. Coinage metals such as silver, gold, and copper, are the most common elements for SERS due to its large SERS enhancement and well-known fabrication methods. Gold and copper have interband absorption in the visible wavelength range, which decreases the maximum SERS intensity^9^. On the other hand, interband absorption of silver is located in the ultraviolet wavelength range, which leads the largest SERS intensity for visible light among these metals^9,10^.

Despite the superior enhancement, silver is difficult to use in acid or heated environment with oxidative gas because of dissolution or oxidation of silver. Graphene, a single atomic carbon layer, has a prominent stability for chemical agents, and its honeycomb lattice completely prevents the penetration of small molecules like hydrogen and water^11^. Therefore, graphene can be useful for a prominent protecting layer of nanostructured silver surfaces for SERS. Besides, graphene has only 2.3% absorption from near infrared to visible range^12^, minimizing the loss of SERS signals from silver.

Composites of nanostructured silver and graphene for SERS have been widely reported using various types of graphene materials such as graphene oxide^13,14^, reduced graphene oxide^15–17^, mechanically exfoliated graphene from bulk graphite^18,19^, and graphene grown by chemical vapor deposition (CVD)^19–22^. Although there are a lot of works on SERS using graphene, there are no reports to focus on the effectiveness of graphene as a protective layer for the plasmonic metal surfaces. Here we studied the chemical stability of graphene in the SERS substrate, consisting of silver nanostructures and single-crystal monolayer graphene^23,24^. Since the SERS for physisorbed molecules on graphene/plasmonic metal has been referred to as graphene-mediated SERS (G-SERS)^19^, we call our developed SERS substrate G-SERS substrate. It is found that the G-SERS substrate showed SERS even in concentrated hydrochloric acid (35–37%) and in heated air up to 400 °C, which is unattainable by the bared silver SERS substrates. The enhancements of Raman signals were observed not only for the protective graphene but also rhodamine 6G (R6G) molecules adsorbed on the graphene, indicating G-SERS can be used for the molecular sensing. Unstable peaks due to nonuniform molecular adsorption, structural change of molecules induced by laser, and metal-molecular interaction are usually observed on the bared silver SERS substrate but G-SERS substrate does not show such behavior which enables us to measure stable and reliable Raman spectra. The high chemical stability of G-SERS can be utilized for new types of SERS techniques such as molecular detections at high temperatures or in extreme acidic conditions.

## Results and Discussion

**Figure 1:**
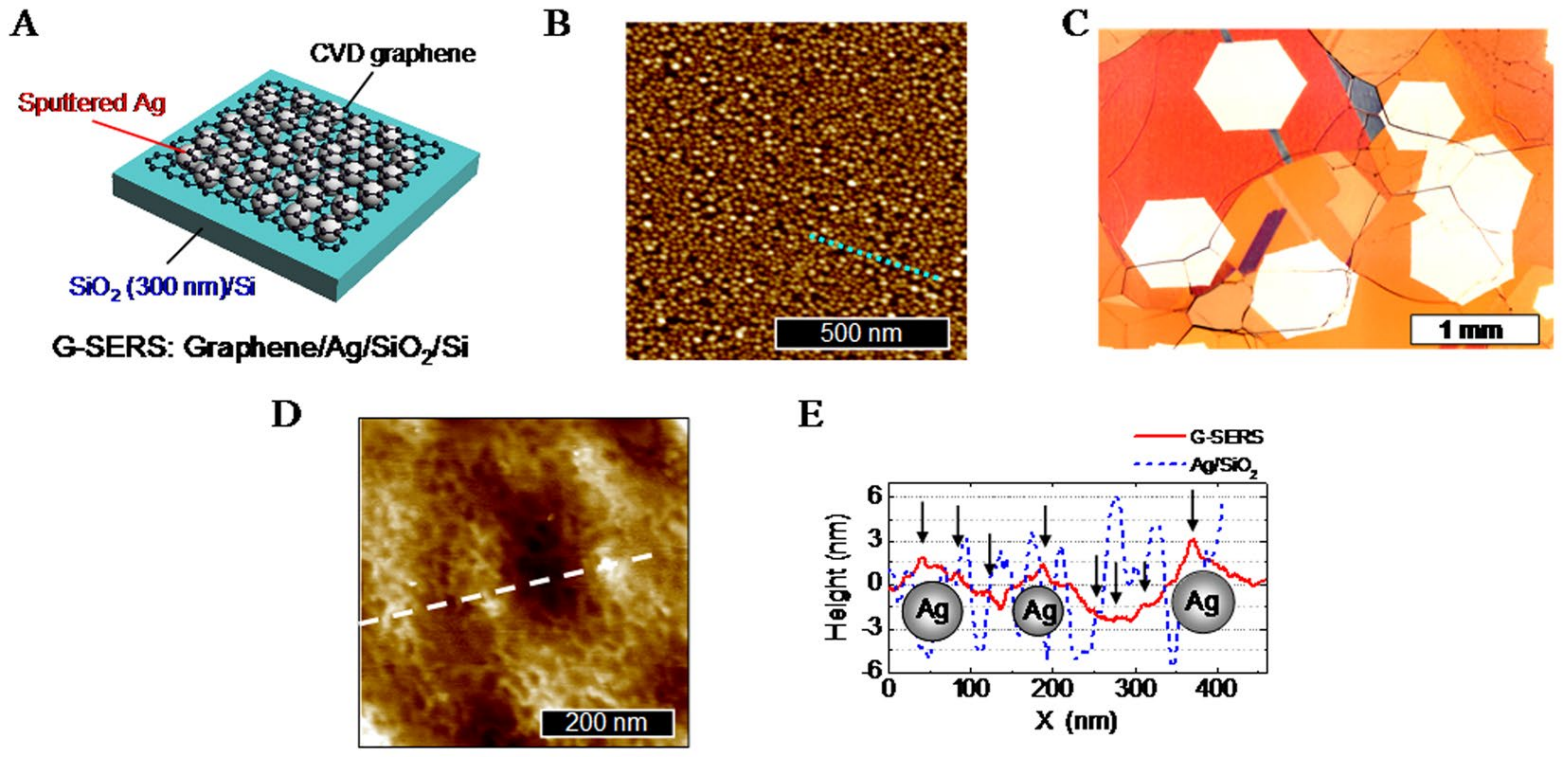
Structure and morphology. (**A**) Schematic illustration of the G-SERS. (**B**) AFM image of sputtered silver on SiO_2_/Si. The deposited thickness of silver (measured by quartz oscillator) was ~2 nm. (**C**) CVD graphene grown on Cu. (**D**) AFM image of the G-SERS surface. (**E**) AFM line profiles along the broken lines in (**B**) and (**E**).

**Figure 2:**
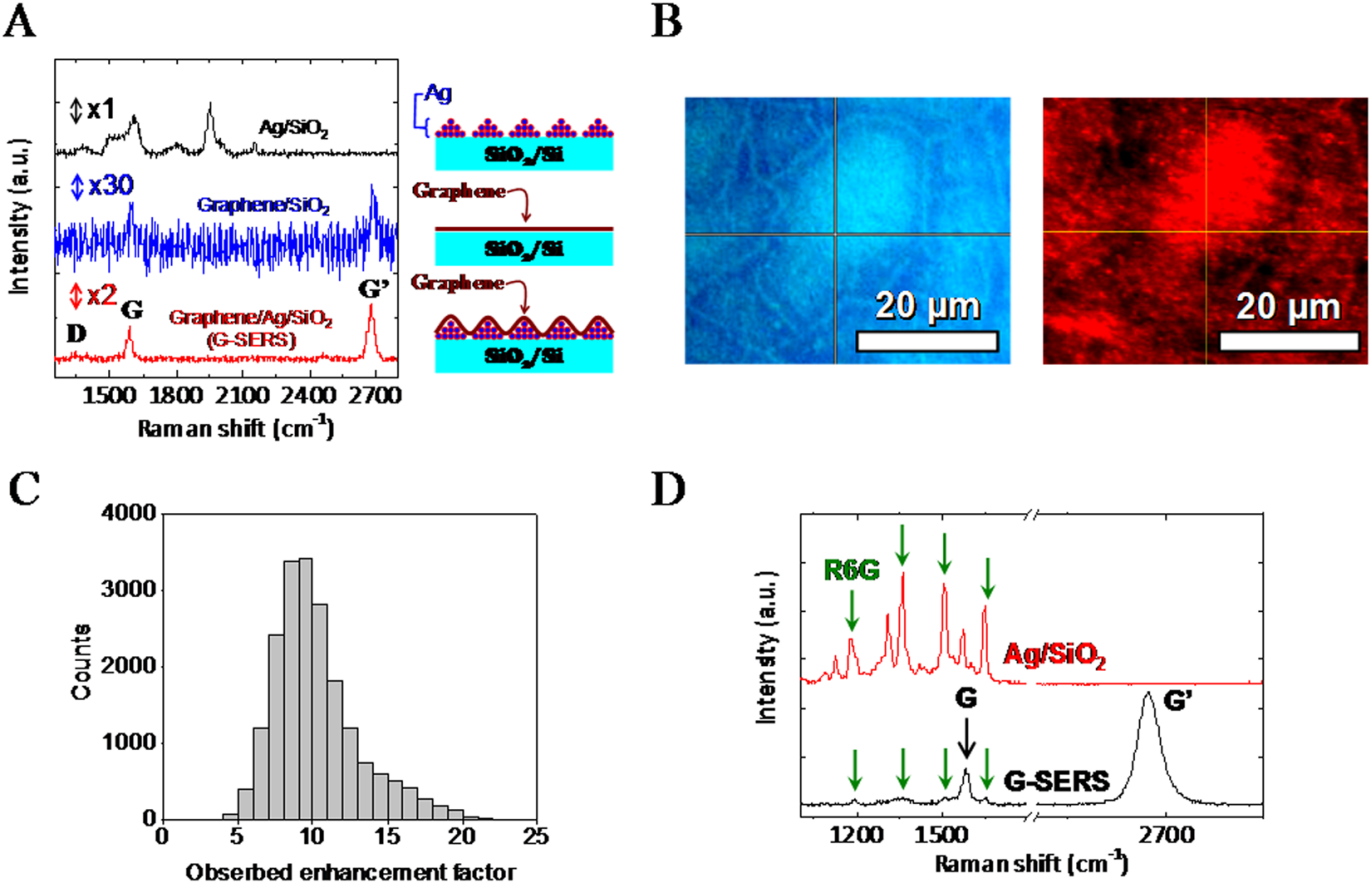
Enhancement of Raman peaks by G-SERS substrate. (**A**) Raman spectra of Ag/SiO_2_/Si (black), graphene/SiO_2_/Si (blue), and graphene/Ag/SiO_2_/Si (red). The number located above each spectrum shows magnifications for the intensity scale. Schematic illustrations for each sample are also shown. (**B**) Optical microscope image (left) and G’ peak intensity map (right) of G-SERS. (**C**) Histogram of the observed enhancement factor of G’ peak intensity. (**D**) Raman spectra of R6G/G-SERS (black) and R6G/Ag/SiO_2_/Si (red).

Figure 1A shows a schematic illustration of the G-SERS substrate. The structure of the G-SERS substrate can be described as graphene/Ag/SiO_2_/Si, where the graphene as a protective layer was prepared by CVD and transfer process, and the silver as plasmonic metal was deposited by sputtering. Figure 1B is an atomic force microscope (AFM) image of the as-deposited silver on a SiO_2_/Si. The sputtering resulted in the formation of nanoparticles with the average height of 6.4 ± 0.7 nm which is considered as the average diameter of the nanoparticles. Figure 1C is an optical microscope image of CVD graphenes on Cu after oxidation at ~200 °C for visualizing graphene. The hexagonal structure corresponds to a single crystal domain of graphene^25,26^, and the size of the domains was approximately 1 mm in diameter. The present CVD graphene is monolayer which is confirmed by Raman spectrum shown in Fig. 2A (discussed later). Figure 1D shows an AFM image of the G-SERS surface. Figure 1E shows AFM line profiles along the broken lines in Fig. 1B and D. In the AFM line profile of the G-SERS (Fig. 1E), we can see large protrusions (3~5 nm) and small protrusions (a few sub-nanometers), as are pointed by arrows. The small protrusions come from the web structure as seen in Fig. 1D, and would be wrinkles of the CVD graphene. The large protrusions are caused by underlying silver particles since the height has similar order to that of the Ag nanoparticles on SiO_2_ (Fig. 1E). The slight decrease of the protrusions in height and density were observed after transfer of graphene (Fig. 1E), indicating bridging of graphene on Ag nanoparticles and aggregation of Ag nanoparticles during the transfer process. Thus, the graphene has a wavy structure along with silver surface. In this configuration, the distance between graphene and silver becomes short, leading to strong enhancement for adsorbed molecules on the graphene^27^.

Figure 2A shows Raman spectra of Ag/SiO_2_/Si (upper), graphene/SiO_2_/Si (middle), and G-SERS substrate (lower). To compare peak intensities, all spectra were taken at the same conditions (laser power: 24 μW, exposure time: 1s). The number located above each spectrum shows the magnification for the intensity scale. The spectrum of Ag/SiO_2_ shows various intense peaks indicating SERS, although these peaks are not reproducible in peak position, width, and intensity (Fig. S1). These non-reproducible peaks are due to carbonaceous impurities adsorbed on silver surface. The carbonaceous impurities initially come from gas molecules from the ambience where the sample was exposed (i.e. in the sputtering chamber^28^ and the Raman measurement chamber (air)). The structure of the adsorbed carbonaceous species can be changed by laser heating^29,30^, which is called photocarbonization^19,29^. The photocarbonization can be seen in Fig. 1SA, and possible peak origins in the Fig. 1SA are summarized in Table S1.

The Raman spectrum of the G-SERS shows G’, G, and negligible small D peaks at around 2680, 1590, and 1345 cm^−1^, respectively. The larger intensities of G’ than G, and small D peak (G/D peak intensity ratio is ~20, see Fig. S2 and Table S2) indicate a monolayer graphene with high crystallinity^31–33^. The G’ and G peaks are also observed in the graphene/SiO_2_ but the intensities are very low compared to the G-SERS substrate. Contrary to Ag/SiO_2_/Si, the spectrum of G-SERS shows steady peaks whereby more stable and reliable SERS can be achieved.

Figure 2B shows an optical microscope image (left) and the G’ peak intensity map (right) of G-SERS. There is a spatial variation in G’ peak intensity, probably due to the variation of microscopic surface structure as shown in the AFM image (Fig. 1D). Figure 2C is the histogram of the observed enhancement factor of G’ peak, which is extracted from the mapping data (Fig. 2B). The enhancement was observed over the entire surface of G-SERS, and the maximum and average enhancement factors were ~24.7 and 10.2 ± 0.6. Figure S6C shows the average Raman spectra of Ag/SiO_2_/Si, graphene/SiO_2_/Si, and G-SERS. The enhancement of the G’ peak was obviously observed in the average spectra, ensuring that SERS enhancement occurs on the entire surface of G-SERS. Figure 2D shows Raman spectra of R6G/G-SERS and R6G/Ag/SiO_2_ in air. The R6G specimen was prepared by dropping and drying 30 μl of R6G/DI solution (10 nM) on each substrate. Prominent Raman peaks of R6G at 1180, 1361, 1507, and 1647 cm^−1^ for R6G/Ag/SiO_2_ and 1195, 1361, 1506, and 1652 cm^−1^ for R6G/G-SERS are observed, in agreement with previous reports^34–36^. Slight peak shifts in the R6G peaks for the G-SERS substrate would be due to the different charge transfer effect between R6G and silver or graphene. The peaks located at 1584 and 2673 cm^−1^ are G and G’ peak, respectively. The other peaks in R6G/Ag/SiO_2_ cannot be assigned, but would be from carbonaceous impurities as mentioned above. In contrast, there are no additional peaks in G-SERS, indicating that G-SERS can be suitable for molecular sensing.

The enhancement mechanism for molecules on G-SERS has been already discussed in a previous work^19^. Xu *et al*. revealed that monolayer graphene shell on Au nanospherical dimer enhances the localized electromagnetic fields rather than decrease^19^. The enhancement mechanism would be same also in our G-SERS since the structure of G-SERS is basically same. The relatively small enhancement for R6G on the G-SERS would be due to structural change of Ag nanoparticles during the process of graphene transfer (Fig. 2B,D, and E).

**Figure 3:**
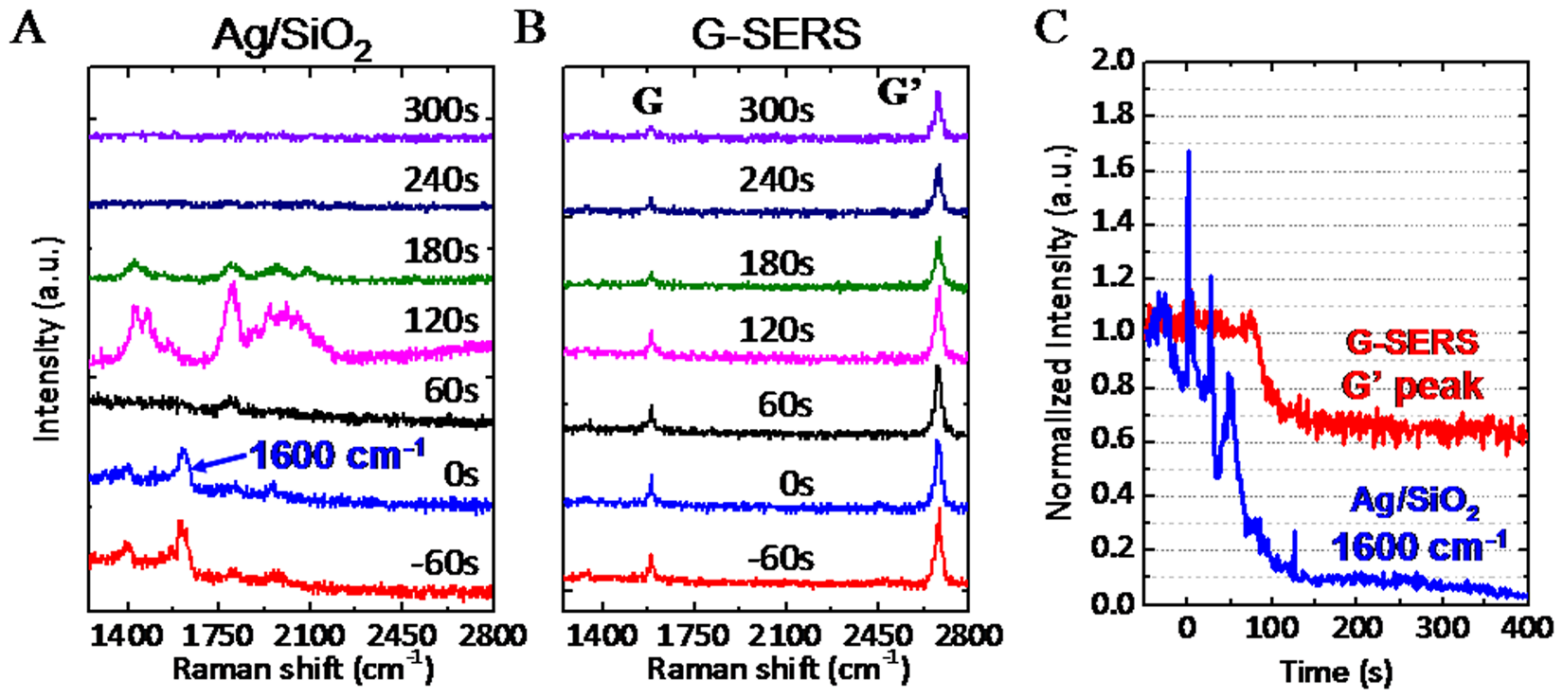
*In situ* HCl test of G-SERS. Time-resolved surface-enhanced Raman spectra of (**A**) Ag/SiO_2_/Si and (**B**) G-SERS at 60 s interval in diluted HCl solution. The concentration of the HCl was 1 × 10^−5^ M. (**C**) Normalized intensities at 1600 cm^−1^ (blue) in (**A**) Ag/SiO_2_/Si and of G’ peak (red) in (**B**) G-SERS with respect to time after immersing in HCl.

To examine chemical stability, the G-SERS substrate was immersed in hydrochloric acid (HCl) with continuously taking Raman spectra. Figure 3A and B show the time-resolved surface-enhanced Raman spectra of Ag/SiO_2_/Si and G-SERS at 60s interval, respectively. The concentration of the HCl was 1 × 10^−5^ M. The SERS peaks in Ag/SiO_2_/Si were disappeared at 60s, but were recovered at 120 s, and then finally completely disappeared at 240s. To understand this behavior, the immersed Ag/SiO_2_/Si surface was observed by AFM, where the size of the silver nanoparticles became larger (Fig. S3C). The size change of the silver particles would be due to silver chloride formation by reaction between HCl and silver nanoparticles^37^, which can also be confirmed by X-ray photoelectron spectroscopy (Fig. S4). The chemical reaction caused the variation in SERS intensities and finally lost enhancement ability of the silver nanoparticles. On the other hand, all SERS peaks in G-SERS were stable after immersing in HCl (Fig. 3B).

Figure 3C shows normalized intensities at 1600 cm^−1^ in Ag/SiO_2_/Si and of G’ peak in G-SERS with respect to time after immersing in HCl. The intensity of G’ peak initially decreases by 40%, and then keep constant, while the peak intensity of 1600 cm^−1^ in Ag/SiO_2_/Si is completely disappeared. Since the average enhancement factor is ~10 (Fig. 2C), the G’ peak was still enhanced nevertheless the decrease by HCl. The decrease in G’ peak would be due to the change of doping level in graphene by HCl^38^. These results indicate that graphene in the G-SERS substrate works as a protective layer which prevents the chemical reaction between silver and HCl. It is also noted that the G-SERS substrate shows SERS after immersing in concentrated HCl (35~37%), indicating its high chemical stability.

To show a potential of G-SERS for practical applications, we focused on the chemical reaction which requires HCl. Normal Ag SERS substrate cannot be used in such chemical reactions, although monitoring chemical reaction is one of the SERS application^39^. As a demonstration for the G-SERS, we measured tertiary butyl chloride (TBC) which was synthesized by tertiary butyl alcohol (TBA) and HCl.

Figure S5 shows Raman spectra of TBA and TBC on G-SERS and SiO_2_/Si substrate. For TBA, a Raman peak at 749.7 cm^−1^ was observed, corresponding to C-C-O (C_3_C-O) symmetric stretching mode^40^ in the molecule. The peak intensity of C-C-O stretching was 2.4 times higher on G-SERS than that on SiO_2_/Si. For TBC, the peak position of C-C-O was shifted to 745.8 cm^−1^, which corresponds to C-Cl stretching mode^41^ in the molecule. The peak intensity of C-Cl stretching was 1.8 times higher on G-SERS than that on SiO_2_/Si. The observed enhancement factors are relatively small, which would be due to weak adsorption of TBA and TBC onto the graphene.

Since the peak shift from C-C-O to C-Cl stretching was clearly observed by G-SERS, G-SERS has enough potential to monitor such chemical reaction. For the practical application for monitoring chemical reactions by G-SERS, its enhancement ability has to be improved, for example, by designing the structure of nanoparticles^39^.

**Figure 4:**
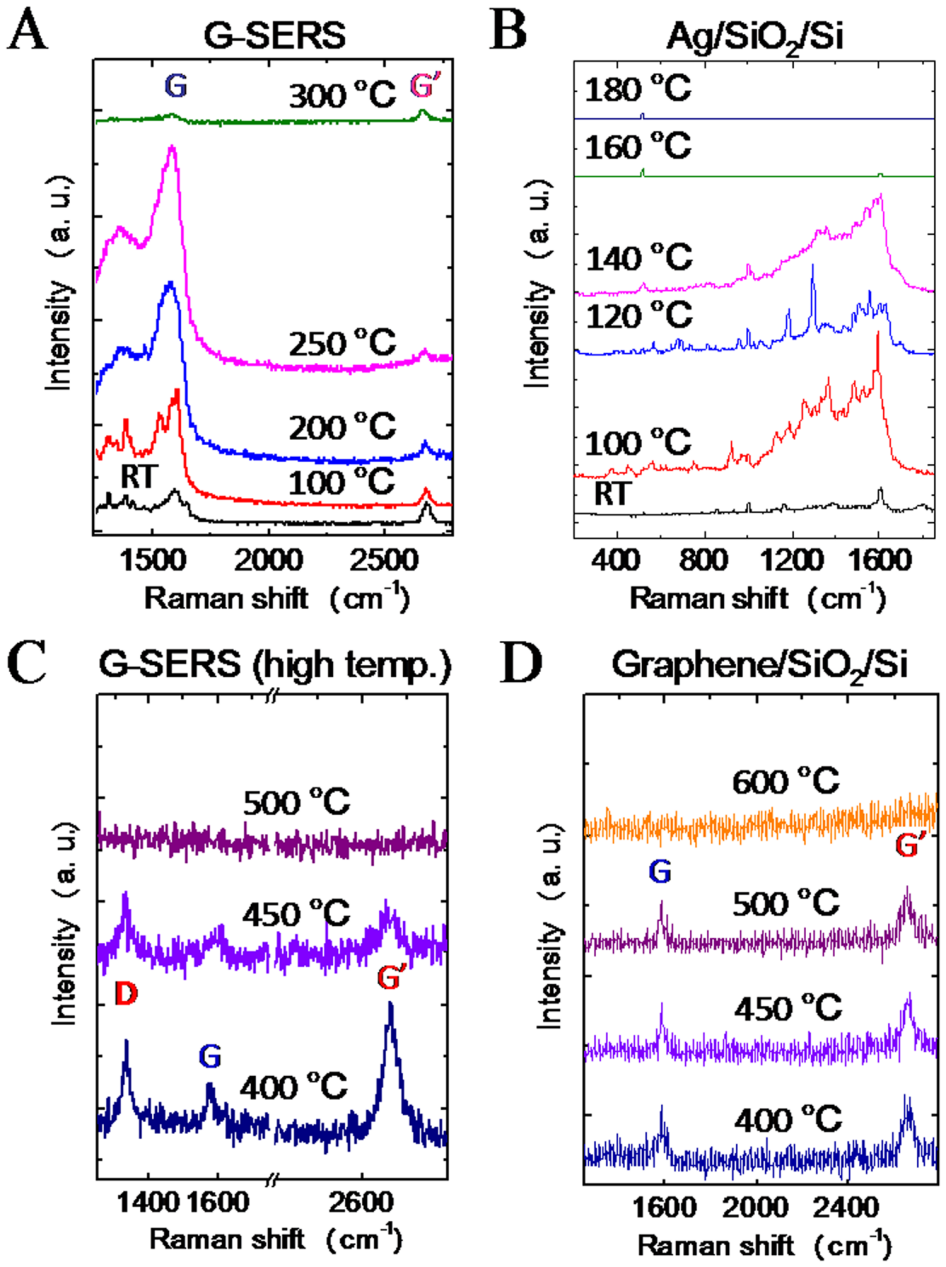
Thermal stability of G-SERS substrate. *In situ* Raman spectra of (**A**) G-SERS and (**B**) Ag/SiO_2_/Si for low temperature heating (<300 °C) in air. *In situ* Raman spectra of (**C**) G-SERS and (**D**) graphene/SiO_2_/Si for high temperature heating (>400 °C) in air.

Subsequently, thermal durability of G-SERS was examined. Figure 4A and B show *in situ* Raman spectra of G-SERS and Ag/SiO_2_/Si at elevated temperatures in air, respectively. These spectra are taken under the same conditions (laser power: 24 μW, exposure time: 5s), and are shown on the same intensity scale. The G-SERS sample shows several large peaks around the G peak at lower than 250 °C (Fig. 4A). The peaks would be from residual polymethyl methacrylate (PMMA) which was used as a supporting layer for the transfer process of graphene. These PMMA peaks were greatly enhanced at 100, 200, and 250 °C, and were disappeared at 300 °C. The disappearance of the peaks would be explained by the complete removal of PMMA residue by oxidation. In the case of Ag/SiO_2_/Si, the SERS was observed until 140 °C, and then disappeared at 160 °C. The temperature of 160 °C is much lower than that the SERS was disappeared in G-SERS. We measured Ag/SiO_2_/Si by X-ray photoelectron spectroscopy (XPS) before and after heating at 200 °C for 15 min (Table S5), and found that the silver was oxidized after the heating. Thus, heating around 200 °C causes oxidation of silver nanoparticles, resulting in the loss of SERS in Ag/SiO_2_/Si. In the G-SERS, graphene is valid as a protective layer for preventing oxidation of silver, resulting in SERS at high temperatures. In addition, G-SERS has uniform spatial distribution in SERS intensity for the mild temperature range (100–140 °C) as shown in Fig. S11, which is an advantage for SERS applications.

To validate the protective function of graphene for long heating duration time, we measured Raman spectroscopy of benzoic acid (BA) dissolved in isopropyl alcohol (IPA) on Ag/SiO_2_/Si and G-SERS. To give clear difference for the long time stability in SERS, Ag/SiO_2_/Si and G-SERS were heated at 150 °C in air up to 63 hours (h).

Figure S8B shows the dependence of the peak intensity of BA at ~1000 cm^−1^ on heating duration time. The intensity of BA on Ag/SiO_2_/Si was drastically decreased in the first 1 h, and kept nearly constant until 30 h, and completely disappeared after 63 h. The intensity of BA on G-SERS was fluctuated for the first 2 h, and reached maximum at 30 h, and decreased to be nearly half of the initial count (~1000) but existing. To examine the reason of the intensity changes in Fig. S8B, we have performed XPS and AFM. XPS analysis revealed that oxidation of Ag particles nearly completed within 1 h, and it does not proceed significantly from 1 to 63 h (Fig. S8C) in Ag/SiO_2_/Si. AFM analysis revealed that the aggregation of Ag particles proceeds gradually by belonging the heating duration time. The speed of aggregation of Ag particles is slower in G-SERS than Ag/SiO_2_/Si (Fig. S9), which provides long time stability in SERS for G-SERS substrates.

Figure 4C and D show *in situ* Raman spectra of G-SERS for higher temperatures (>400 °C) and graphene/SiO_2_/Si at each temperature in air, respectively. In the G-SERS substrate (Fig. 4C), the D peak was appeared at 400 °C and the peak intensities of D, G, and G’ peaks were decreased at 450 °C, and eventually all peaks were disappeared at 500 °C, indicating the loss of graphene. In the graphene/SiO_2_/Si (Fig. 4D), G and G’ peaks were observed until 500 °C but disappeared at 600 °C. To understand the loss of graphene at lower temperature in G-SERS, we compared the G’ peak positions and temperature as shown in Fig. S7. The G’ peak positions were monotonically decreased both for G-SERS and graphene/SiO_2_/Si, while the G’ peaks in G-SERS show lower position at almost all temperature. The redshift of G’ peak indicates that graphene is stretched by heating^42–44^. The larger redshifts in G-SERS than graphene/SiO_2_/Si indicate that the graphene in G-SERS is initially stretched by following the structure of Ag particles (Fig. 1D). Since the stretched graphene is more reactive for oxygen radicals than the flat graphene^45^, the initial stretch would result in loss of graphene at lower temperatures. It is also noted that the G’ peak at 400 °C in G-SERS (Fig. 4C) has ~2.8 times larger peak intensity than that in normal graphene, indicating that the enhancement still occurs at 400 °C. This result shows that the present G-SERS substrate can be useful for high temperature SERS applications.

## Conclusions

In conclusion, a chemically stable G-SERS substrate was successfully fabricated by using single crystal monolayer CVD graphene. We revealed that the G-SERS substrate has high tolerance with HCl and high temperature oxidation up to 400 °C. The maximum and average observed enhancement factors of G’ peaks were ~24.7 and 10.2 ± 0.6, and the enhancement was observed over the entire surface. The enhancement factor can be further increased by adjusting the preparation process of silver nanoparticles. The fabrication method of the G-SERS substrate including CVD graphene is a scalable process. Thus, the chemically stable G-SERS substrate has a practical and feasible application and its high chemical stability can provide new type of SERS technique such as molecular detections at high temperatures or in extreme acidic conditions.

## Methods

Silver nanoparticles were deposited by dc magnetron sputtering (Quorum Technologies, Q300T D) at room temperature with a silver target of 99.99% purity in argon atmosphere. The deposition pressure and rate were 1 Pa and ~1.3 Å/s. The deposited thickness (assuming uniform film structure) was measured by a quartz oscillator in the sputtering chamber to control the particle size of silver. The deposited thickness was used to control the particles size. The deposited thickness of silver ranging from 2 to 6 nm shows similar order enhancements in Raman signals for 532 nm wavelength.

Graphene was grown by a custom-made atmospheric pressure chemical vapor deposition (CVD) system^23,24^. The purities of H_2_ and Ar gasses are 99.99999% (7N), and 99.9999% (6N), respectively. The chamber pressure has been kept to be near atmospheric pressure (0.123~0.128 MPa) during the CVD process. 100 μm-thick commercial Cu foils (Nilaco, #113321) with the size of ~2 cm × 10 cm were used as catalytic substrates for the growth. The grown graphene was transferred onto arbitral target substrate with a conventional method, which uses a spin-coated polymethyl methacrylate (PMMA) support layer, ammonium peroxodisulfate for etchant of Cu, and the mixture of acetone, isopropyl alcohol, and methyl isobutyl ketone for removal of PMMA^46,47^.

Surface morphologies of samples were microscopically observed using an atomic force microscope (AFM, Bruker, Multimode) in the peak force tapping mode and an optical microscope (Keyence, Digital Microscope VHX-5000). Surface elemental analyses were performed by XPS (ULVAC-PHI, PHI 5000 VersaProbe II) under a base pressure of ~6 × 10^−8^ Pa and a monochromatized AlKα (1486.6 eV) X-ray source. Raman spectra were recorded with a Raman microscope (Renishaw, InVia) at a wavelength of 532 nm in the backscattering geometry. *In situ* heating experiments were carried out in the temperature controllable stage (Linkam, THMS 600).

## Electronic supplementary material

Supplementary Information
