# Supplementary material for: Chemical Stability of Graphene Coated Silver Substrates for Surface-Enhanced Raman Scattering
Source: Sci Rep. 2017 Nov 1;7:14851. doi: 10.1038/s41598-017-14782-2 (PMC5665989; doi:10.1038/s41598-017-14782-2)
Supplement: Supplementary file 1 — Supplementary Information [file 41598_2017_14782_MOESM1_ESM.pdf]

Supplementary Information

Chemical Stability of Graphene Coated Silver Substrates for Surface-Enhanced Raman Scattering

Seiya Suzuki[*] *and Masamichi Yoshimura*

Graduate School of Engineering, Toyota Technological Institute,

Nagoya, 468-8511, Japan

1. Reproducibility of Raman peaks in Ag/SiO_2/Si and G-SERS

Figure S1 shows the time dependence of Raman spectra on Ag/SiO_2/Si and G-SERS. The Raman spectra at 0s in Figs. S1A and S1B are same as that in Fig. 2A. G and G' peaks were only observed on G-SERS without any change in peak shape, while the Raman peaks on Ag/SiO_2/Si changes with time.

We categorized the nonreproducible peaks on Ag/SiO2 into 5 groups as shown in Table S1. The corresponding vibration modes for each Raman shift range are quoted from "The Handbok of Infrared and Raman Characteristic Frequencies of Organic Molecules", written by D. Lin-Vien et al. (ACADEMIC PRESS, INC.).

All peaks are considered to be due to surface carbon-based adsorbates from the atmosphere through the photocarbonization (R. Cooney et al. Chem. Phys. Lett. 76, 448 (1980); doi.org/10.1016/0009-2614(80)80645-2). On the Ag nanoparticles, peak shifts and new band generation can be seen due to the interaction between the adsorbed molecules and silver as well as the modification of selection rules which is caused by alteration of the symmetry of the vibrational modes. Actually, a peak group of 1, 2, and 3 is often observed in SERS measurement. For instance, Büchel, D. et al. reported that sputtered silver surface which has SERS provides this peak group (Büchel, D. et al. Appl. Phys. Lett. 79, 620 (2001); doi.org/10.1063/1.1389513).

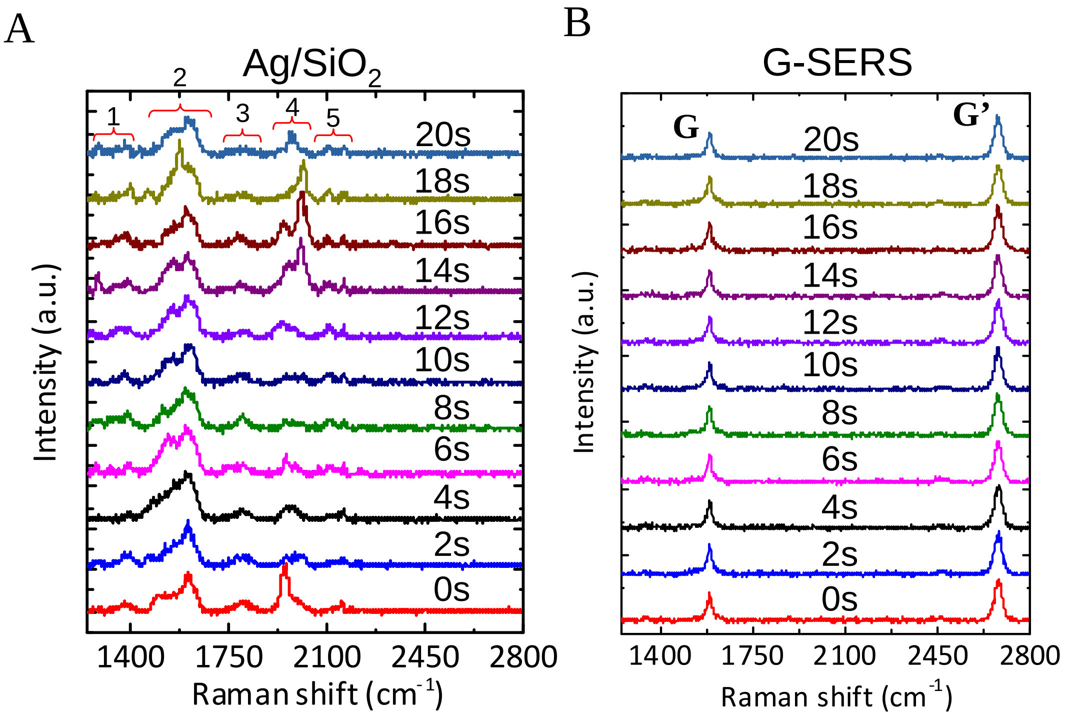

Figure S1. Comparison of the time dependence of Raman spectra of (A) $Ag/SiO_2/Si$ and (B) G-SERS. The spectra at 0s correspond to the spectra in Fig. 2A. Ag/SiO_2 shows non-reproducible peaks but G-SERS does not show any peaks, except those from graphene. Peaks 1 and 2

Table S1: Features of nonreproducible Raman peaks on $Ag/SiO_2/Si$. The nonreproducible peaks were categorized for 5 groups, which are indicated in Fig. S1A. The abbreviations in the Table are deformation (def.), scissoring (sci.), and stretching (str.). The corresponding vibration modes are from "The Handbook of Infrared and Raman Characteristic Frequencies of Organic Molecules", written by D. Lin-Vien et al. (ACADEMIC PRESS, INC.).

Peak group	Ranges (cm^{-1})	Peak position	Intensity	Corresponding vibration modes in each Raman shift range
1	1250-1450	Random	small	CH def., CH rock, α-CH bend, CH_3 def., CH_2 sci., Semicircle str. (aromatic), Ring str. (anthracenes, naphthalenes), OH bend (alcholes), CO_2^- str.
2	1500-1600	Random	large	C=C str.
3	1780-1800	Small changes	small	C=O str.
4	1950-2000	Random	Random	C≡C str. (acetylene gas)<br>(C=C=C out of phase str.: IR)
5	~2100<br>and<br>~2150	Small changes	small	C≡C str., symetric C≡C-C≡C str.,<br>(Alkyl acetylenes,<br>C=C=O out of phase str.: IR)

2. Quality of graphene

Figure S2 shows the Raman spectra of graphene/SiO_2/Si with exposure time for 1 and 10 s. Raman spectrum with exposure time for 10 s was used to analyze Raman peak properties of the graphene. Table S2 shows peak positions, widths, and intensities of D, G, and G' peaks.

The G/D ratio, which indicates quality of graphene, is ~ 20.4. T. J. Lyon et al. recently reported field effect transistor (gFET) of CVD graphene (Appl. Phys. Lett. **110,** 113502 (2017); dx.doi.org/10.1063/1.4978643). The CVD graphene possess the G/D ratio less than 10, and its gFET devices work pretty well (mobility is 2000-4000 cm^2/Vs which is usual value in gFET fabricated by CVD graphene). Thus, we would like to insist our produced CVD graphene is of high quality. It is also noted that we can grow much high quality graphene which possesses the G/D ratio higher than 50 (S. Suzuki et al., Jpn. J. of Appl. Phys. **52,** 125102 (2013); doi.org/10.7567/JJAP.52.125102).

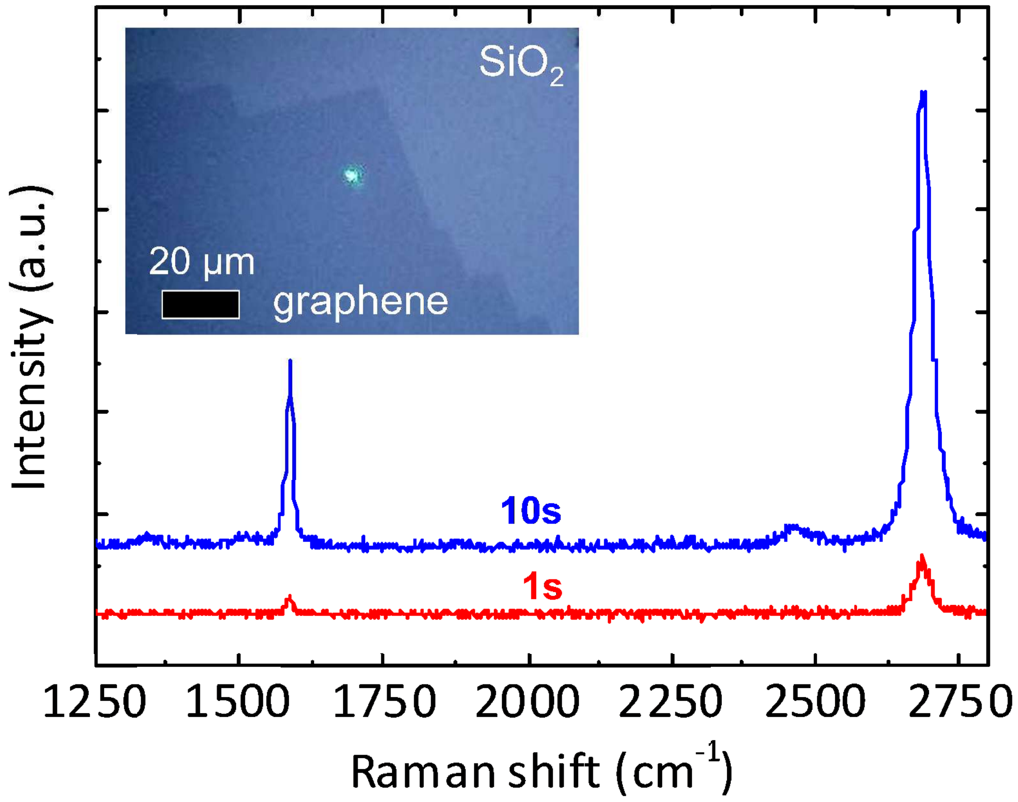

Figure S2. Raman spectra of graphene/SiO_2/Si recorded with exposure time for 1s (red) and 10s (blue). Inset shows an optical image of the graphene/SiO_2/Si. Lower left and upper right areas show graphene and SiO_2 surface, respectively. The green dot at the center in the image is the laser spot where the spectra were taken.

Table S2: Peak positions, widths, and intensities for Raman spectrum of graphene/SiO_2/Si with exposure time for 10s.

Peak	Peak position (cm^{-1})	Peak width (cm^{-1})	Intensity
D	1342.4	37.7	16.8
G	1588.4	13.8	342.2
G'	2686.1	34.9	902.7

3. AFM images of Ag/SiO2 and GSERS before and after immersed in hydrochloric acid

Figures S3A and S3B show AFM images of Ag/SiO_2 and G-SERS before immersed in hydrochloric acid (1×10^{-4} M) for 15 min, respectively. Figures S3C and S3D show AFM images of Ag/SiO_2 and G-SERS after immersed in hydrochloric acid, respectively. The deposited thickness by Ag sputtering was 6 nm for those samples. Figures S3E and S3F compares AFM line profiles of Ag/SiO_2 and G-SERS before and after immersed in hydrochloric acid, respectively.

The AFM results indicated that the surface morphology is changed by hydrochloric acid for Ag/SiO_2 but not G-SERS. The size and the density of naked Ag nanoparticles were increased and decrease by immersing in hydrochloric acid, respectively. Li and Zhu reported that the Ag nanoparticles have high chemical reactivity and become AgCl by reacting with HCl (J. Colloid and Interface Sci. **303,** 415 (2006); doi.org/10.1016/j.jcis.2006.07.059). The changes of the size and the density would be due to coalescence of Ag nanoparticles or the formation of AgCl nanoparticles during the chemical reaction.

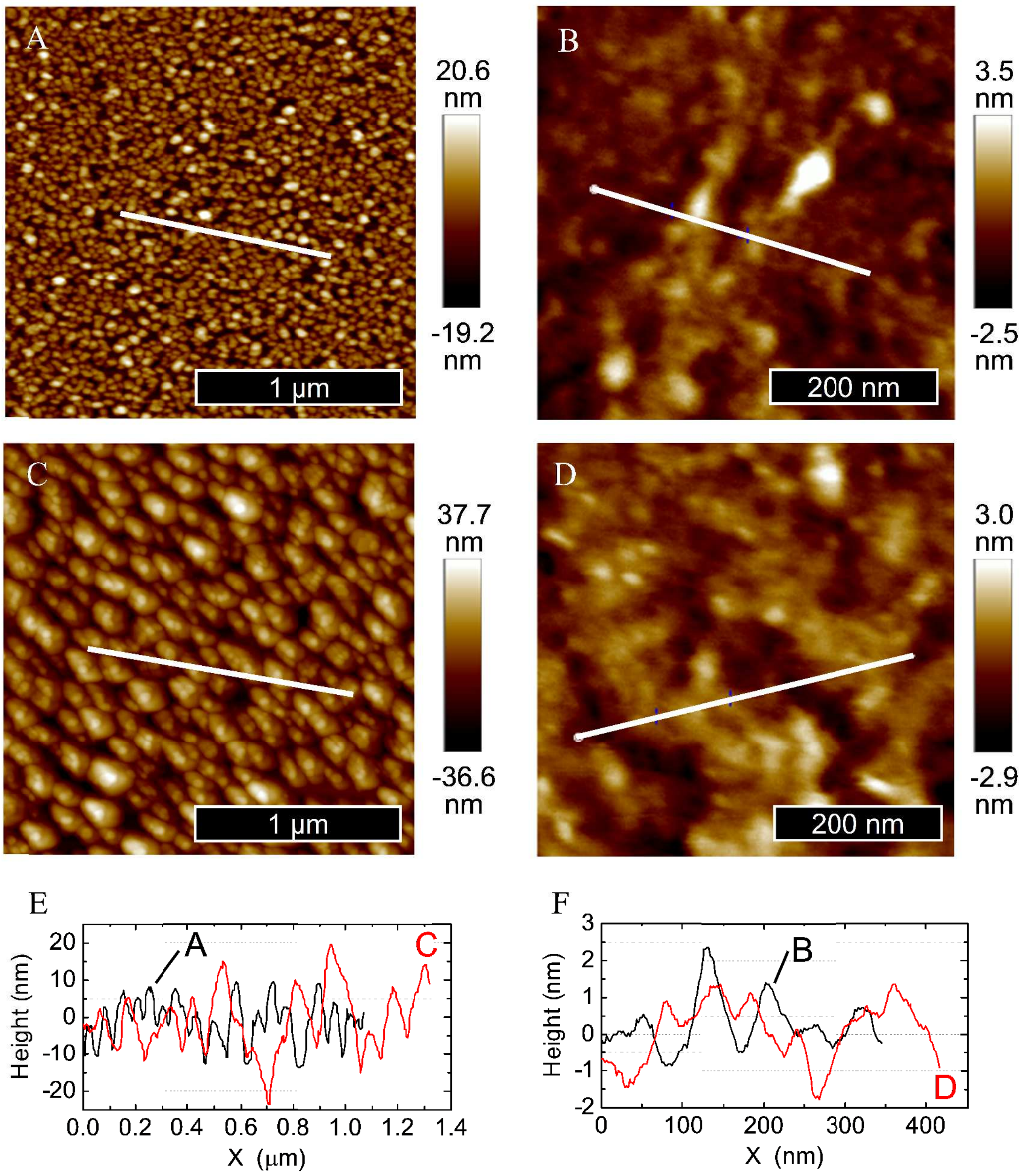

Figure S3: AFM images of (A) Ag/SiO_2 and (B) G-SERS. AFM images of (C) Ag/SiO_2 and (D) G-SERS after immersed in hydrochloric acid (1×10^{-4} M). The deposited thickness by Ag sputtering was 6 nm for those samples. AFM line profiles of (E) Ag/SiO_2 and (F) G-SERS along the solid lines in (A-D).

4. Elemental analysis for reaction between HCl and Ag nanoparticles

We measured Ag/SiO_2/Si samples by X-ray photoelectron spectroscopy (XPS) to determine silver chloride formation by reaction between HCl and silver nanoparticles.

The deposited thickness of the Ag for the sample was 6 nm. XPS spectra for the sample was obtained before and after immersing in HCl (1×10^{-4} M) for 15 min. Binding energy calibration for obtained XPS spectra was performed using the position of the Si 2p peak in SiO_2 at 103.3 eV.

Figure S4A shows representative XPS survey spectra of Ag/SiO_2/Si before and after immersed in HCl. Silicon, Si 2p, at 103.3 eV and oxygen, O1s, at 532 eV were observed and corresponded to the SiO_2 substrate. Carbon, C1s, at 285 eV was observed and corresponded to carbonaceous impurities. Silver, Ag 3d5/2, at 368 eV was observed and corresponded to the sputtered Ag nanoparticles. Figure S4B shows enlarged XPS spectra in Fig. S4A. Cl2p peak was only observed in the spectrum of Ag/SiO_2/Si after immersed in HCl. Figure S4C shows a XPS narrow spectrum of Ag/SiO_2/Si around Cl2p after immersed in HCl. Chlorine, Cl2p3/2 and Cl2p5/2, at 197.9 and 199.5 eV were observed.

Table S3 shows relative atomic ratios to Si calculated from XPS narrow spectra of Ag/SiO_2/Si before and after immersed in HCl. Amount of carbon, oxygen, silver were decreased and a few amount of chlorine was appeared by immersing Ag/SiO_2/Si in HCl. The existence of chlorine ensures AgCl formation by reaction between HCl and Ag nanoparticles as reported previously by Li and Zhu (J. Colloid and Interface Sci. **303,** 415 (2006); doi.org/10.1016/j.jcis.2006.07.059). Decrease of the Ag amount by HCl would be due to the dissolving AgCl to aqueous solution. Decrease of the carbon and oxygen amounts indicate decrease of carbonaceous impurities on the surface, which could be caused by reduce of the catalytic activity of Ag nanoparticles by colorization.

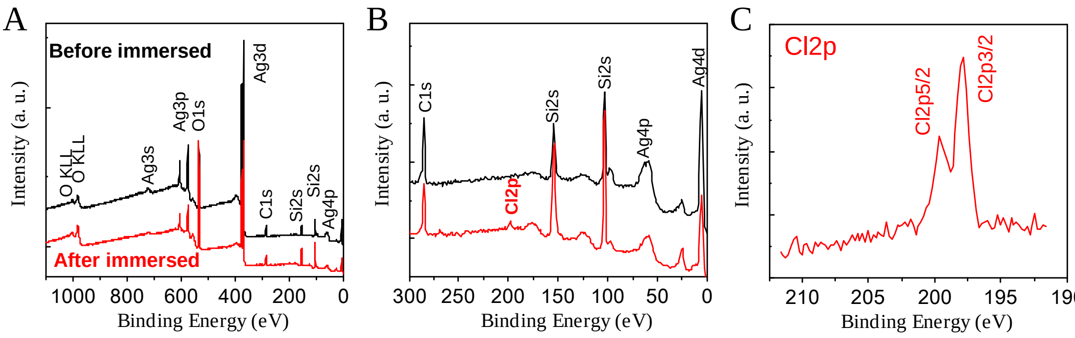

Figure S4: (A) Representative XPS survey spectra of $Ag/SiO_2/Si$ before and after immersed in HCl (1×10^{-4} M) for 15 min. (B) Enlarged XPS spectra in A. Cl2p peak was observed. (C) XPS Cl2p spectrum of $Ag/SiO_2/Si$ after immersed in HCl.

Table S3: Relative atomic ratios to Si calculated from XPS narrow spectra of $Ag/SiO_2/Si$ before and after immersed in HCl. To obtain average values of the atomic ratios, we obtained 3 spectra for each samples.

	before	after
C/Si	0.80±0.01	0.47±0.02
O/Si	2.37±0.02	2.18±0.01
Ag/Si	1.42±0.01	0.43±0.02
Cl/Si	-	0.021±0.001

5. Detection of tertiary butyl alcohol and tertiary butyl chloride by G-SERS

Commercially available tertiary butyl alcohol (TBA) and hydrochloric acid (HCl) were used to synthesize tertiary butyl chloride (TBC). The purities of the TBA and the HCl are more than 99% and 35-37%, respectively. To prepare samples for Raman, 1 ml of TBA (~ 0.01 M) and 1.2 ml of HCl (~0.01M) were mixed and dropped on Ag/SiO_2/Si and G-SERS. Subsequently, a cover glass was placed on the sample, and then Raman spectra were recorded as well as for pure TBA.

Figure S5 shows Raman spectra of TBA and TBC on G-SERS and SiO_2/Si substrate. For TBA, a Raman peak at 749.7 cm^{-1} was observed and corresponded to C-C-O (C_3C-O) symmetric stretching mode (P. K. Kipkemboi et al., Bull. Chem. Soc. Ethiop. **17,** 211 (2003); dx.doi.org/10.4314/bcse.v17i2.61689) in the molecule. The peak intensity of C-C-O stretching was 2.4 times higher on G-SERS than that on SiO_2/Si. For TBC, the peak position of C-C-O was shifted to 745.8 cm^{-1}, which is corresponded to C-Cl stretching mode (D. Lin-Vien et al. "The Handbook of Infrared and Raman Characteristic Frequencies of Organic Molecules", Elsevier (1991).) in the molecule. The peak intensity of C-Cl stretching was 1.8 times higher on G-SERS than that on SiO_2/Si. The observed enhancement factors are relatively small, which would be due to weak adsorption of TBA and TBC onto graphene.

Since the peak shift from C-C-O to C-Cl stretching was clearly observed by G-SERS, G-SERS has potential to monitor such chemical reaction. To have practical application for monitoring chemical reactions by G-SERS, its enhancement ability has to be improved, for example, by designing the structure of nanoparticles (W. Xie et al., J. Am. Chem. Soc. **135,** 1657 (2013); dx.doi.org/10.1021/ja309074a).

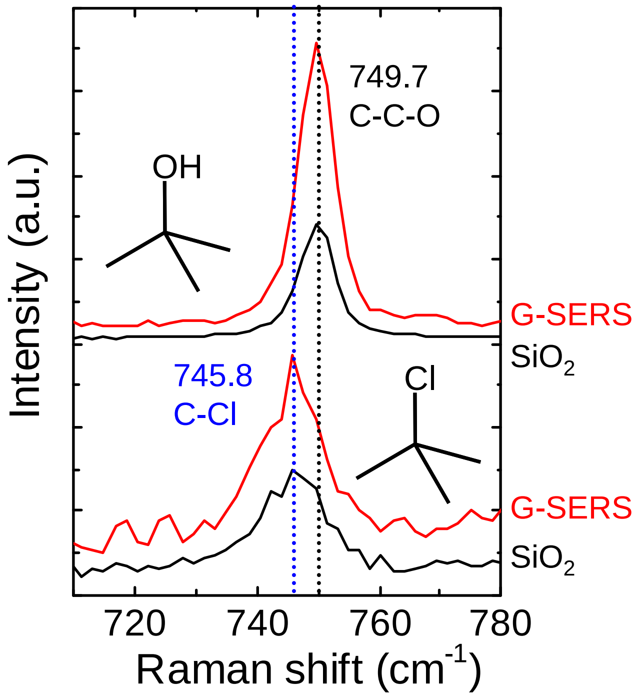

Figure S5: Raman spectra of tertiary butyl alcohol (upper) and tertiary butyl chloride (lower) on G-SERS (red line) and SiO_2/Si (black line) substrate. Intensity scales for tertiary butyl chloride were magnified 7 times to show the shapes of these peaks.

6. EDS analysis of Ag/SiO_2/Si for 200 ºC heating in air

We measured Ag/SiO_2/Si by EDS (Energy dispersive X-ray spectrometry) before and after heating at 200 ºC for 15 min in air but no significant changes were observed (Table S4).

Table S4: Atomic ratios of Ag/SiO_2/Si in % before and after 200 ºC heating in air. The atomic ratios were calculated from EDS spectra.

	RT	200 °C
Si	59.49	59.07
O	30.70	30.60
C	9.52	10.03
Ag	0.29	0.30

7. XPS analysis of $Ag/SiO_2/Si$ for 200 ºC heating in air

Instead of EDS, we performed X-ray photoelectron spectroscopy (XPS) which is a surface sensitive elemental analysis. Table S5 shows relative atomic ratios of $Ag/SiO_2/Si$ to Ag before and after 200 ºC heating in air, which are obtained from XPS results. The oxygen signal contains that from SiO_2. Since the SiO_2 (300 nm) is sufficiently thick, all silicon signals from the SiO_2 layer not the bottom Si. Two times of Si/Ag relative atomic ratio is nearly equal to the O/Ag from the SiO_2. When we subtract the two times of the Si/Ag from the O/Ag, we obtain the O/Ag except oxygen from SiO_2 (the bottom row in Table S5).

It was observed that the O/Ag except oxygen from SiO_2 increases after heating at 200 ºC. Since oxidation of carbon results in gas phase such as CO and CO_2, it is the most likely that the increase of oxygen is mainly due to oxidation of Ag.

Table S5: Relative atomic ratios of $Ag/SiO_2/Si$ to Ag before and after 200 ºC heating in air. The relative atomic ratios were calculated from XPS spectra. The Ag except oxygen from SiO_2 at the bottom row is obtained by subtracting two times of the Si/Ag from the O/Ag.

	RT	200 °C
C/Ag	0.56±0.01	0.74±0.02
O/Ag	1.66±0.02	2.69±0.05
Si/Ag	0.70±0.01	1.1±0.02
O/Ag except oxygen from SiO_2	0.26±0.02	0.49±0.04

8. Raman Mapping of $Ag/SiO_2/Si$ and Graphene/SiO_2/Si

Raman mapping of $Ag/SiO_2/Si$ and graphene/SiO_2/Si were measured here. The Raman mappings were obtained at the same condition as that of Fig. 2B (~2 mW, 0.1s exposure time). It is noted that the laser power was much higher than that in Fig. 2A (24 μW) to reduce the laser exposure duration for the mapping.

Figures S6A and S6B show optical microscope images (left) and peak intensity maps (right) at 1606 cm^{-1} and G' peak of (A) $Ag/SiO_2/Si$ and (B) Graphene/SiO_2/Si, respectively. Figure S6C shows the average spectra of $Ag/SiO_2/Si$, graphene/SiO_2/Si, and G-SERS. A thousand spectra were averaged to obtain these spectra from Figs. S6A, S6B, and Fig. 2B. Among the several intense spectra in the average spectrum of $Ag/SiO_2/Si$, the most intense peak (1606 cm^{-1}) was used for the Raman mapping in Fig. S6A.

The several intense peaks in the $Ag/SiO_2/Si$ (Fig. S6A) are due to surface carbon-based adsorbates from the atmosphere through the photocarbonization (R. Cooney et al. Chem. Phys. Lett. 76, 448 (1980); doi.org/10.1016/0009-2614(80)80645-2), which was mentioned in the answer #4 (Fig. S1A and Table S1). The optical microscope image of $Ag/SiO_2/Si$ shows uniform contrast while the intensity map shows random distribution. The inhomogeneity spectral broadening due to photo-induced carbonization gives difficulty for quantitative evaluation.

From the average spectra (Fig. S6C), it is apparent that the graphene/SiO_2/Si does not have any extra peak, except G and G', indicating uniformity of the graphene in crystal quality.

The average spectra of G-SERS shows broaden peaks around D (1340 cm^{-1}) and G (1585 cm^{-1}) peak positions, which is different from Fig. 2A. The difference would be due to photocarbonization with the high power laser exposure, and residual PMMA, and defects and strain in the graphene induced by the wavy structure. The lower laser power would suppress photocarbonization and the residual PMMA can be removed by oxidation process which is demonstrated in Fig. 4A.

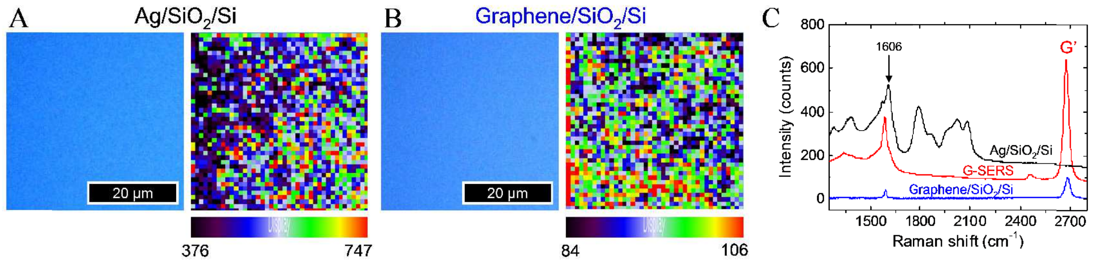

Figure S6: Optical microscope images (left) and peak intensity maps (right) at 1606 cm^{-1} and G’ peak of (A) $Ag/SiO_2/Si$ and (B) $Graphene/SiO_2/Si$, respectively. Color bars at the bottom of the peak intensity maps indicate corresponding colors for peak intensity. (C) Average Raman spectra of (A), (B), and Fig. 2B, as $Ag/SiO_2/Si$, $Graphene/SiO_2/Si$, and GSERS, respectively.

9. Temperature dependence of G' peak for G-SERS and graphene/SiO_2/Si

Figure S7 shows the temperature dependence of the G' peak position for the G-SERS and the graphene/SiO_2/Si. G' peaks for both samples show monotonic decrease tendency with increasing temperature. The G' peak position is sensitive to strain [W. Wang et al., J. Mater. Sci. Mater. Electron **27,** 3888 (2016); doi 10.1007/s10854-015-4238-y, I. Calizo et al., Nano Lett. **7,** 2645 (2007); doi 10.1021/nl071033g, T. M. G. Mohiuddin et al., Phys. Rev. B **79,** 205433 (2009); doi: 10.1103/PhysRevB.79.205433] so that the redshift of G' peak indicates that graphene is stretched. The G' peak position in the G-SERS is lower than that in the graphene/SiO_2/Si for almost all temperature. This indicates the graphene in G-SERS is stretched by following surface structure of Ag nanoparticles. The Raman peaks of graphene in G-SERS were completely disappeared at 500 °C, which is lower temperature that in graphene/SiO_2/Si, indicating the attribution of the inherent stress in graphene which is induced by the surface structure of Ag nanoparticles.

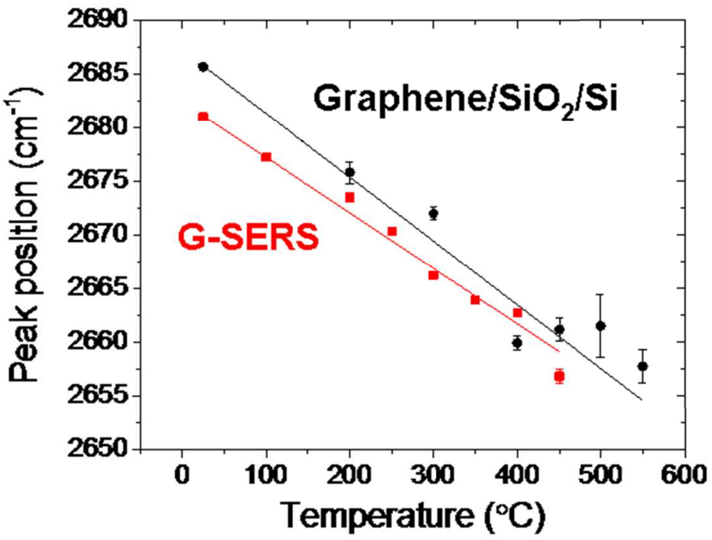

Figure S7: Temperature dependence of the G' peak position for the G-SERS and the graphene/SiO_2/Si. The red and black lines are fitted lines to the experimental data.

10. Long term protective function of graphene

To examine the long term protective function of graphene, we had Raman measurements of benzoic acid (BA) dissolved in isopropyl alcohol (IPA) at different heating duration time in air. To give clear difference for the long time stability in SERS, we heated Ag/SiO_2/Si (Ag-SERS) and G-SERS at 150 ºC in air till 63 hours (h). Raman spectroscopy, XPS, and AFM were used to study the protective function of graphene.

The deposited thickness was 2.2 nm for both Ag-SERS and G-SERS for this measurement. 0.1 M BA/IPA solution was prepared. A 25 μL drop of the BA solution was deposited on the samples, and then a cover glass was immediately placed on it for suppressing evaporation of the solution. Subsequently, Raman spectra of BA on Ag-SERS and G-SERS were recorded through the cover glass under same conditions (laser wavelength 532 nm, laser power: 24 μW, exposure time: 10s). After obtaining Raman spectra, the samples were rinsed by pure IPA for 5 min and then were heated at 150 ºC in air. After the heating, the samples were cooled down to room temperature and Raman spectra of BA were obtained in a same method as mentioned above. This measurement was repeated till 63 h for heating.

Figure S8A shows Raman spectra of BA on Ag-SERS and G-SERS before and after heating at 150 ºC for 63 h. Representative Raman peaks of BA at 1003 and 1602 cm^{-1} can be identified in the spectra for both Ag-SERS and G-SERS before heating. BA peaks were difficult to be observed on bare SiO_2/Si due to the low signal at the measurement condition. When we increased the laser power to be 120 μW, BA peaks at 1002 and 1605 cm^{-1} were observed. The peak intensity of the 1002 cm^{-1} peak was around 70 which can be a reference for observed enhancement factors for Ag-SERS and G-SERS. After the heating for 63 h, BA peaks were disappeared in Ag-SERS but remained in G-SERS.

Figure S8B shows the dependence of the peak intensity of BA at ~ 1000 cm^{-1} on heating duration time. The intensity of BA on Ag-SERS was drastically decreased in the first 1 h, kept nearly constant until 30 h, and completely disappeared after 63 h. The intensity of BA on G-SERS was fluctuated for the first 2 h, reached maximum at 30 h, and decreased to be nearly half of the initial count (~1000) but existing. The observed enhancement factor of G-SERS after 63 h is higher than 7.

To examine the reason of the intensity changes in Fig. S8B, we have performed XPS. An Ag-SERS sample for the XPS measurement was prepared without BA deposition to avoid contamination leading exploit intrinsic effects of heating in air at 150 ºC for long time. Figure S8C shows the dependence of surface atomic ratios of the

Ag-SERS on heating duration time at 150 ºC, which are obtained by XPS results. The atomic ratios of O/Ag and Si/Ag were increased but C/Ag was decreased by heating especially the first 1 h. The decrease of the C/Ag indicates that carbon contaminants become less by heating. Since escape depth of photoelectrons is few nanometers range, removal of very thin carbon contaminants leading the increase of the signal from SiO_2 substrate surface, which is consistent with the increase of the Si/Ag. Thus O/Ag includes the oxygen signal from SiO_2. The green plots in Fig. S8C were obtained by subtracting 2 times of the Si/Ag, which gives O/Ag ratio without oxygen from SiO_2. The O/Ag was increased by factor of ~1.5 in 1 h and slight increased (1.06 times) after that. The O/Ag signal also contain signals form carbon contaminates but it would be not likely happen since the total carbon amount becoming less. Therefore, the increase of the O/Ag is due to oxidation of Ag by heating at 150 ºC. The oxidation of Ag nearly completed within 1 h, and it does not proceed significantly after that. Since the SERS intensity in Ag-SERS was drastically decreased in the first 1 h (Fig. S8B), the oxidation of Ag would lead it. It is noted that Ag was not fully oxidized with heating at 150 ºC and its structure may have core-shell structure of AgO_x/Ag.

Since the SERS intensity changes after 1 h (Fig. S8B) cannot be explained by the XPS results, we measured surface morphology of Ag-SERS and G-SERS by AFM. Figures S9A-C show AFM images of Ag-SERS as deposited and after heating at 150 ºC for 30 and 63 h. Aggregation of Ag particles was observed after heating for 30 h (Fig. S9B). It was observed that the aggregation proceeds gradually by belonging the heating duration time (Fig. S9C). After the heating for 63 h, density and number of isolated particles were decreased and increased than 30 h, indicating decrease of hot spots for SERS. The decrease of hot spots by thermal aggregation would be the reason why the decrease of SERS intensity from 1 to 63 h.

Figures S9D and S9E show AFM images of G-SERS after heating 150 ºC for 30 and 63 hours, respectively. It was observed that particle structures of Ag exist after heating for both. The particle size was increased from 30 to 63 h, indicating aggregation of the particles by heating. Comparing the G-SERS (Fig. S9E) and the Ag-SERS (Fig. S9C) after 63 h, the G-SERS has higher density of particles. This indicates that graphene layer prevents aggregation of Ag particles for long time heating at mild temperature (150 ºC). Thus, the higher SERS intensities of G-SERS than Ag-SERS after heating were provided by graphene which suppresses aggregation and oxidation [A. S. Kousalya et al., Corrosion Science **69,** 5 (2013); doi.org/10.1016/j.corsci.2012.12.014] of Ag particles. The very high intense SERS after heating for 30 h (Fig. S8B) would be because particle size and spatial arrangement of particles for hot spots were

unintentionally optimized for SERS with 532 nm excitation laser by aggregation. This result indicates SERS intensity can be increased by mild heating (150 ºC) in air.

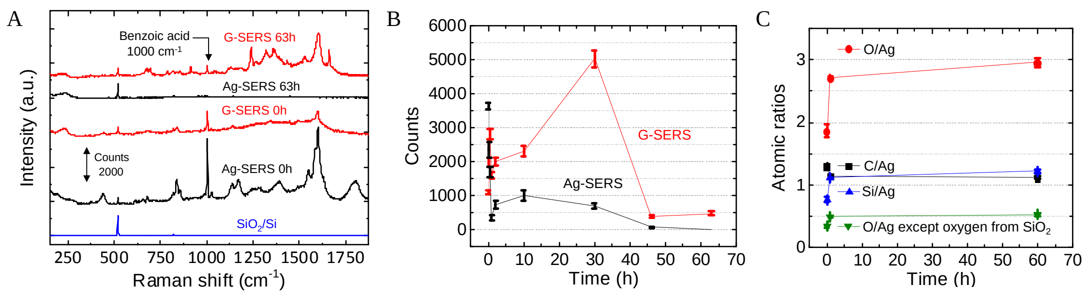

Figure S8: (A) Raman spectra of benzoic acid (0.1 M in IPA) on Ag-SERS (black) and G-SERS (red) before and after heating at 150 ºC for 63 hours. Raman spectrum of benzoic acid on SiO_2/Si (blue) was shown as a reference for the Raman peak enhancements. (B) Dependence of the Raman peak intensity of benzoic acid at 1000 cm^{-1} (ring breathing mode) on heating duration time. (C) Dependence of surface atomic ratios of Ag-SERS on heating duration time at 150 ºC. The surface atomic ratios were obtained by XPS.

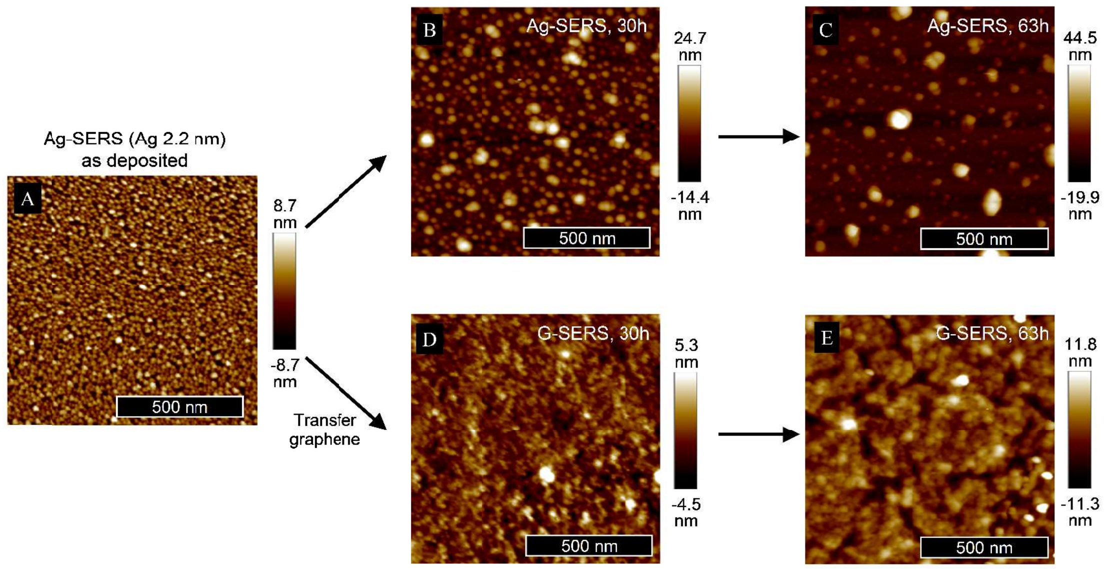

Figure S9: AFM images of Ag-SERS (A) as deposited and after heating at 150 ºC for (B) 30 and (C) 63 hours, and G-SERS after heating at 150 ºC for (D) 30 and (E) 63 hours.

11. Spatial distribution of Raman intensities of G-SERS and Ag-SERS at high temperatures.

The spatial distribution of SERS intensity in Ag/SiO_2/Si (Ag-SERS) and G-SERS at high temperature has been discussed in this section.

Ag nanoparticles for the Ag-SERS and G-SERS were prepared by sputtering with deposited thickness of 2.2 nm. 0.1 M BA/IPA was dropped and dried onto the Ag-SERS and the G-SERS. Raman mapping for 30 × 30 μm area with a 1 μm step were obtained at RT, 100 ºC, 120 ºC, and 140 ºC in air. The laser wave length, the laser power, and the exposure time were 532 nm, 2 mW, and 0.1 s, respectively. All mappings were recorded within 10 min to avoid aggregation of Ag particles by long time heating which is mentioned in the previous answer.

Figures S10A and S10B show average Raman spectra of Ag-SERS and G-SERS at different temperature, respectively. The average Raman spectra were obtained by averaging all spectra (961 spectra) in the Raman mappings. While the peak shapes and intensities in the spectra of G-SERS were not changed significantly by temperatures, the peak intensities in the spectra of Ag-SERS were wholly decreased by increasing temperatures. As discussed in the previous answer, 1 hour heating at 150 ºC in air oxidizes Ag particles resulting in decrease of SERS intensities. In addition, aggregation of Ag particles was not clear by heating at 150 ºC for 1 hour (Fig. S12). Therefore, we conclude that the decrease of SERS from 100 to 140 ºC is also due to oxidation of Ag particles.

Figure S11 shows spatial distribution of Raman intensities of G-SERS and Ag-SERS at different temperature. Significant peaks in the average spectra (Fig. S10A and S10B) were used to make these Raman maps. The peak at 1585 cm^{-1} in G-SERS mainly consists of G peak and aromatic vibration in graphene and benzoic acid, respectively, and the peak at 1600 cm^{-1} in Ag-SERS is mainly from benzoic acid. The size and the step of the Raman intensity maps are 30 × 30 μm^2 and 1 μm, respectively. The color scales for all maps are ranged from zero to average intensity in the Raman intensity of each peak. In the Ag-SERS, low intensity area was increasing by increasing temperature. The distribution change would indicate that the degree of Ag oxidation was slightly different from the spatial position, which would be related to uniformity of surface such as Ag particles size and impurity amount. In contrast, it is difficult to find the significant distribution change for both the G’ peak and the peak at 1585 cm^{-1} in G-SERS. This result reinforces that G-SERS has prominent stability for high temperature.

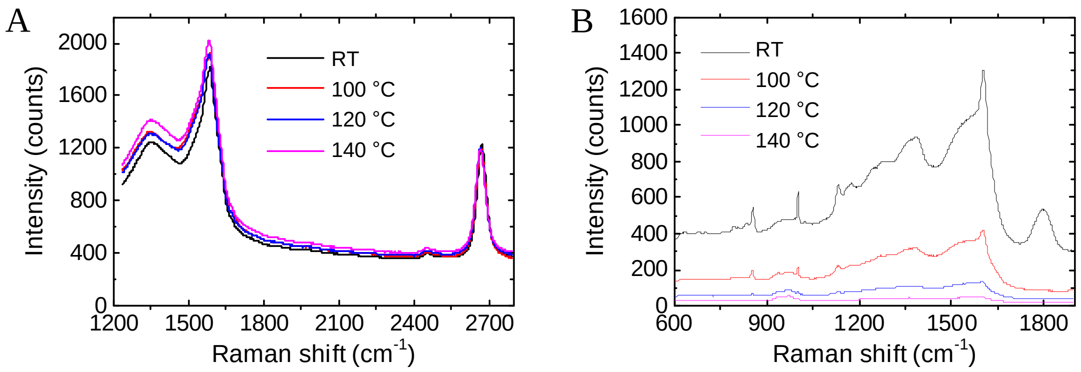

Figure S10: Average Raman spectra of (A) G-SERS and (B) Ag-SERS at high temperatures. Benzoic acid was deposited onto for both samples.

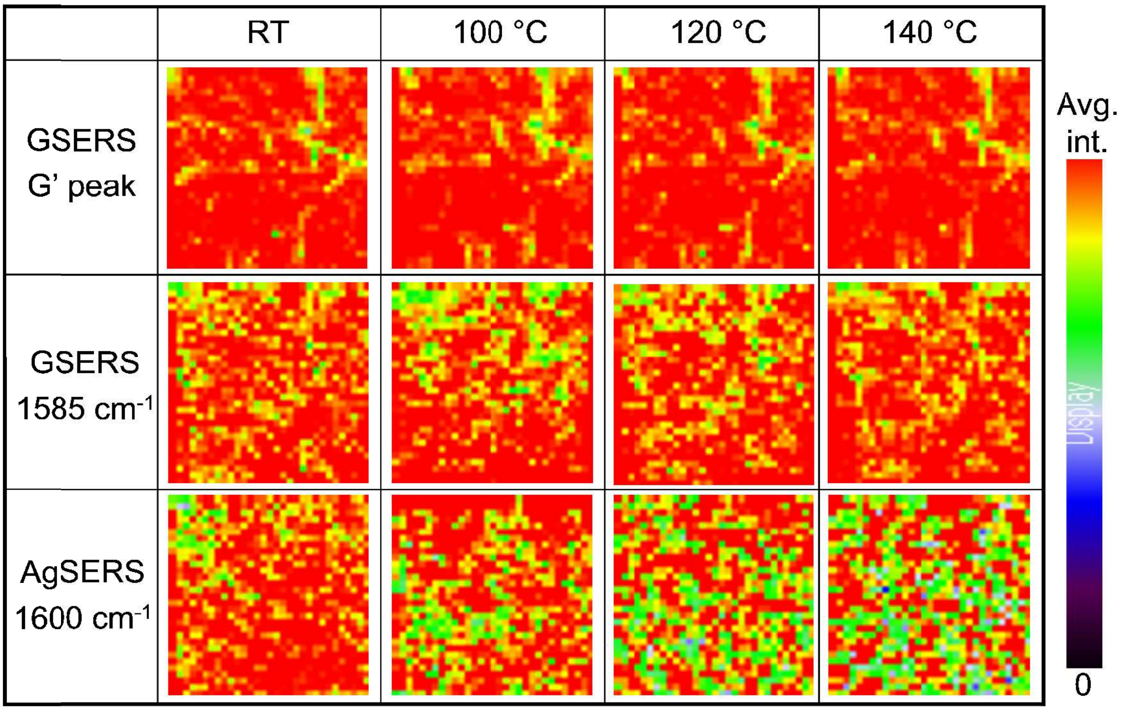

Figure S11: Spatial distribution of Raman intensities of G-SERS and Ag-SERS at different temperature. The size and the step of the Raman intensity maps are 30 × 30 μm^2 and 1 μm, respectively. The color scales for all maps are ranged from zero to average intensity in the Raman intensity of each peak.

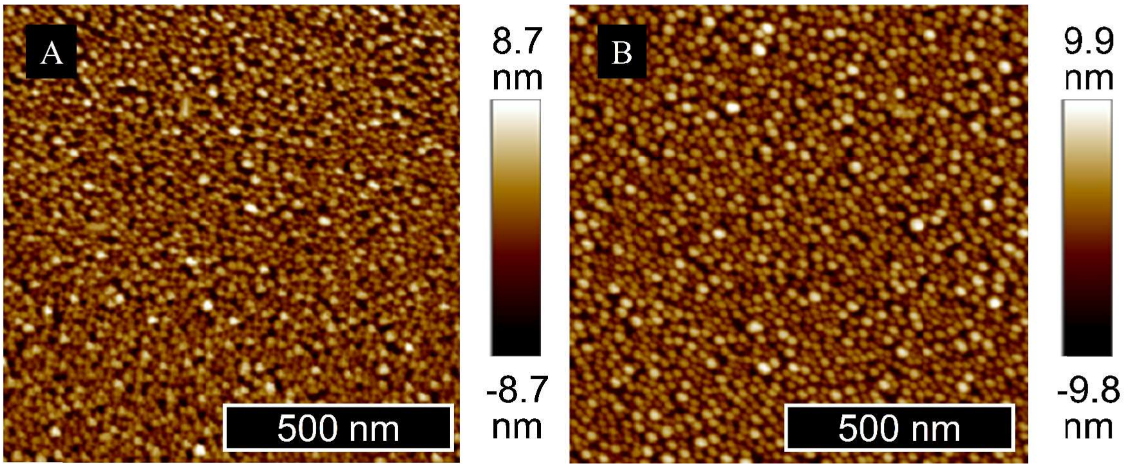

Figure S12: AFM images of Ag-SERS (A) as deposited and (B) after heating at 150 ºC for 1 hour.
